# The potential of cystatin C as a predictive biomarker in pulmonary hypertension

**DOI:** 10.1186/s12890-023-02595-1

**Published:** 2023-08-26

**Authors:** Anqi Duan, Zhihua Huang, Zhihui Zhao, Qing Zhao, Qi Jin, Lu Yan, Yi Zhang, Xin Li, Sicheng Zhang, Meixi Hu, Luyang Gao, Chenhong An, Qin Luo, Zhihong Liu

**Affiliations:** 1 Center for Respiratory and Pulmonary Vascular Diseases, Department of Cardiology, Fuwai Hospital, National Clinical Research Center for Cardiovascular Diseases, National Center for Cardiovascular Diseases, Chinese Academy of Medical Sciences and Peking Union Medical College, No.167 Beilishi Rd, Xicheng District, Beijing, 100037 China; 2 Department of Cardiology, Shanghai Institute of Cardiovascular Disease, Zhongshan Hospital, Fudan University, Shanghai, China; 3 Center for Critical Care Medicine, Sichuan Provincial People’s Hospital, Chengdu, China

**Keywords:** Biomarker, Pulmonary hypertension, Cystatin C, Renal function, Risk prediction

## Abstract

**Background:**

Cystatin C is a novel biomarker to identify renal dysfunction and cardiovascular risk.

**Objective:**

The aim of this study was to investigate the role of cystatin C in non-invasive risk prediction in a large cohort of patients with pre-capillary pulmonary hypertension (PH).

**Method:**

We retrospectively analyzed pre-capillary PH patients with available cystatin C and hemodynamic data derived from right heart catheterization.

**Results:**

A total of 398 consecutive patients with confirmed pre-capillary PH were recruited from Fuwai Hospital between November 2020 and November 2021. Over a median duration of 282 days, 72 (18.1%) of these patients experienced clinical worsening. Cystatin C levels significantly correlated with cardiac index (*r* = -0.286, *P* < 0.001), mixed venous oxygen saturation (*r* = -0.216, *P* < 0.001), and tricuspid annular plane systolic excursion (*r* = -0.236, *P* < 0.001), and high cystatin C levels independently predicted a poor prognosis after adjusting potential confounders in different models (all *P* < 0.05). A three-group non-invasive risk model was constructed based on the combined assessment of the cystatin C and WHO-FC using dichotomous cut-off value. Those patients with higher cystatin C (≥ 1.0 mg/L) and a worse WHO-FC experienced the highest risk of endpoint occurrence. The predictive capacity of this model was comparable to that of an existing invasive risk stratification model (area under curve: 0.657 vs 0.643, *P* = 0.619).

**Conclusions:**

Cystatin C levels were associated with disease severity and prognosis in patients with pre-capillary PH. A combination of high cystatin C and advanced WHO-FC identifies patients at particularly high risk of clinical deterioration.

**Supplementary Information:**

The online version contains supplementary material available at 10.1186/s12890-023-02595-1.

## Introduction

Pulmonary hypertension (PH) is a life-threatening hemodynamic condition characterized by elevated pulmonary arterial pressure and pulmonary vascular resistance, leading to exercise intolerance, right heart failure (HF), and potentially fatal outcomes. The World Health Organization (WHO) classifies PH into five groups. Group 1 includes pulmonary arterial hypertension (PAH), group 2 includes PH associated with left heart disease, group 3 includes PH associated with lung disease and/or hypoxia [1], group 4 includes chronic thromboembolic PH (CTEPH) and other pulmonary artery obstructions, and group 5 includes PH with unclear and/or multifactorial mechanism. Right heart catheterization (RHC) is considered the gold standard for diagnosis and regular follow-up [2, 3]. However, its invasiveness and cost have prompted exploration into non-invasive biomarkers that could identify PH patients at higher risk. These biomarkers, reflecting vascular dysfunction, metabolism, oxidative stress, inflammation, or secondary organ damage (e.g., kidney, liver, gastrointestinal tract), hold promise for routine use in PH centers and clinical trials.

Cystatin C, a cysteine protease inhibitor that plays a role in vascular biology, is one of the promising biomarkers in risk prediction of PH. Several studies have detected elevated levels of cystatin C in PH patients, and higher levels have been linked to an increased risk of mortality [4–7]. One plausible explanation for the observed association between serum cystatin C and adverse outcomes is its ability to provide a precise measure of glomerular filtration rate (GFR), which serves as a crucial indicator of renal insufficiency and is closely linked to poor prognosis [8]. Traditionally, GFR estimation has relied on equations incorporating serum creatinine and/or cystatin C. Cystatin C, as a biomarker, offers distinct advantages in assessing renal function. Unlike creatinine, cystatin C is unaffected by muscle mass, age, sex, or dietary intake, making it a more reliable measure of GFR [9]. Moreover, cystatin C has been found to be more sensitive in detecting early renal dysfunction and subtle changes in renal function [10]. Recently, a novel equation was recommended by the American Society of Nephrology and the National Kidney Foundation. This equation, known as the 2021 Chronic Kidney Disease Epidemiology Collaboration (CKD-EPI) eGFRcr and 2021 CKD-EPI eGFRcr-cys, notably excludes the race variable and has demonstrated superior accuracy compared to previously established equations [9–11]. Another explanation for the association between cystatin C and PH is that cystatin C is also associated with oxidative stress and inflammation in the pulmonary vasculature, and its effect on cardiovascular disease outcomes has been shown to be independent of estimated GFR [12, 13].

Despite the potential benefits of using cystatin C in risk assessment of pre-capillary PH patients, there is limited clinical evidence available. In this study, we aimed to explore the role of cystatin C in non-invasive risk prediction of pre-capillary PH.

## Methods

### Study population

Consecutive patients diagnosed with PH according to 2022 ESC/ERS guidelines [3] in our center at Fuwai Hospital in Beijing, China, from November 2020 to November 2021 were screened for enrollment. Patients who underwent serum cystatin C and creatinine tests, as well as RHC during hospitalization were included in the analysis. Exclusion criteria were: (1) under the age of 18; (2) classified as post-capillary PH; (3) previous kidney transplantation. Demographic data, physical examination results, the presence of comorbidities, 6-min walking distance (6MWD), World Health Organization functional class (WHO-FC) and biochemistry measurements were recorded. Baseline blood samples were collected from a peripheral vein after overnight fast, and biochemical parameters were measured using standard laboratory methods. The study was conducted in accordance with the Declaration of Helsinki and the protocol was reviewed by the Ethics Committee of Fuwai hospital [Approval number: 2020–1364], with written informed consent obtained from all participants.

### Echocardiography

All patients underwent standard transthoracic echocardiography conducted by experienced cardiologists as previously detailed [14]. The left ventricular ejection fraction was calculated using the Simpson biplane method. The modified Bernoulli equation was employed to determine the systolic pulmonary artery pressure (PASP). The tricuspid annular plane systolic excursion (TAPSE) was measured in the apical 4-chamber view.

### RHC

Experienced pulmonary vascular physicians performed RHC on all patients through the femoral or internal jugular vein. During the procedure, the following hemodynamic parameters were recorded: right atrial pressure (RAP), mean pulmonary artery pressure (mPAP), and pulmonary arterial wedge pressure. The mixed venous oxygen saturation (SvO_2_) was also measured. Cardiac output was calculated using the Fick method, and cardiac index, total pulmonary resistance, and pulmonary vascular resistance were determined using standard formulas [15].

### eGFR equations and stages

Baseline GFR was estimated using different equations shown as follows: 2021 CKD-EPI eGFR_cr_ [9], 2021 CKD-EPI eGFR_cr-cys_ [9], 2012 CKD-EPI eGFR_cr-cys_ [10], 2012 CKD-EPI eGFR_cys_ [10], 2009 CKD-EPI eGFR_cr_ [16], Cockcroft-Gault [17], and Modification of Diet in Renal Disease (MDRD) equations [18]. eGFR stages were determined according to Kidney Disease Improving Global Outcomes guidelines.

### Risk stratification

The Swedish/COMPERA (Comparative, Prospective Registry of Newly Initiated Therapies for Pulmonary Hypertension) method, an abbreviated version of the risk stratification scheme recommended by PH guidelines [19] was performed to categorize patients into low, intermediate or high-risk groups using the following variables: WHO-FC, 6MWD, N-terminal pro-brain natriuretic peptide (NT-proBNP) level, RAP, cardiac index and SvO_2_ [20]. Each variable was graded according to the cut-off values proposed in guidelines (Table S1). The sum of all grades was divided by the number of available variables, and then rounded to the nearest integer.

### Follow-up

The composite endpoint of clinical worsening was defined as any of the following events: a) death, b) rehospitalization for HF or deterioration of PH (characterized by worsening WHO-FC or a ≥ 15% reduction in 6-min walk distance from baseline), and c) escalation of targeted therapy (specifically, the introduction of parenteral prostacyclin analog therapy). Participants’ outcomes were tracked through their in-hospital or outpatient medical records, and telephone visits were conducted until the occurrence of outcome events or the end of the study (December 12th, 2022). Time to clinical worsening was calculated as the duration from the date of the baseline serum cystatin C measurement to the occurrence of specific outcomes. These endpoint events, indicative of clinical worsening, were meticulously evaluated by two experienced clinicians. In the event of any discrepancies in the assessment, consensus was reached through discussion with senior supervisors (QL and ZHL).

### Statistical analysis

Continuous data were expressed as mean (± standard deviation) or median (interquartile ranges) according to distribution, and categorical data were expressed as frequencies (percentages). Baseline characteristics were compared using two-tailed t test or Wilcoxon rank sum test (continuous variables) and Pearson chi-square test or Fisher exact test (categorical variables).

Univariate Cox regression analysis was performed and Wald chi-square test statistic was calculated. Univariate receiver operating characteristic (ROC) curves were created, and area under the curve (AUC) was used to compare the discrimination of different renal function parameters and eGFR equations for clinical worsening. Considering the limited number of events and a relatively large number of variables, different multivariate Cox proportional hazards models were built, corrected for potential confounders. The adjusted variables for multivariate analysis were restricted by univariate *P* value < 0.10.

Additionally, a restricted cubic spline with three knots was performed to identify the dose–response relationship between the predictors and clinical worsening. The Kaplan–Meier method was used to generate the survival curve, and the log‐rank test was performed. Spearman correlation analysis was performed to examine correlations between variables. Statistical significance was set at *P* < 0.05. All statistical tests were performed using the statistical package for the social science (SPSS) software (version 22.0; IBM SPSS Statistics, IBM Corp., Armonk, NY, USA) and R (version 4.0.5, R Foundation for Statistical Computing, Vienna, Austria).

## Results

### Baseline characteristics

From November 2020 to November 2021, 540 consecutive patients with PH were enrolled in our study. The diagnoses of pre-capillary PH were confirmed via RHC in 413 patients according to the latest guidelines [3]. After excluding patients who were under 18 years old or had undergone kidney transplantation, baseline cystatin C was collected in 398 patients (Fig S1).

The majority of the patients were female (63.8%) and were classified as WHO FC II (50.2%) or III (44.2%). Congenital heart disease-associated PAH (31.2%) and idiopathic PAH (27.4%) were the most common subtypes of PH. The patients had a mean age of 48.04 ± 16.52 years and a mean 6MWD of 409.44 ± 85.38 m. 53 patients (13.3%) exhibited kidney dysfunction, defined as 2021 CKD-EPI_cr-cys_ eGFR < 60 mL/min/1.73m^2^. The baseline characteristics of included participants are summarized in Table 1.

**Table 1:** Demographic, clinical, echocardiographic and hemodynamic characteristics of study population

Variables	Total (*N* = 398)	CW (*n* = 72)	No-CW (*n* = 326)	*P*-value
Age, years	48.04 ± 16.52	51.92 ± 15.90	47.18 ± 16.55	**0.027**
Female, n (%)	254 (63.8)	38 (52.8)	216 (66.3)	**0.031**
BMI, kg·m^−2^	23.61 ± 4.97	23.08 ± 4.35	23.73 ± 5.10	0.321
WHO-FC, n (%)				**0.001**
I or II	215 (54.0)	26 (36.1)	189 (58.0)	
III or IV	183 (46.0)	46 (63.9)	137 (42.0)	
6MWD, m	409.44 ± 85.38	404.71 ± 76.43	410.16 ± 86.83	0.754
Hypertension, n (%)	90 (22.6)	17 (23.6)	73 (22.4)	0.823
Diabetes Mellitus, n (%)	39 (9.8)	10 (13.9)	29 (8.9)	0.197
Dyslipidemia, n (%)	47 (11.8)	8 (11.1)	39 (12.0)	0.839
Etiologies, n (%)				
IPAH	109 (27.4)	18 (25.0)	91 (28.0)	0.620
CHD-PAH	124 (31.2)	22 (30.6)	102 (31.3)	0.891
CTD-PAH	20 (5.0)	3 (4.2)	17 (5.2)	0.944
CTEPH	73 (18.3)	17 (23.6)	56 (17.2)	0.202
Others ^a^	72 (18.1)	12 (16.7)	60 (18.4)	0.729
*Echocardiography*				
LVDd, mm	41.36 ± 7.57	41.31 ± 6.80	41.38 ± 7.74	0.942
RVDd, mm	33.95 ± 7.54	36.90 ± 8.09	33.30 ± 7.26	** < 0.001**
LVEF, %	64.86 ± 7.10	63.60 ± 7.96	65.14 ± 6.87	0.096
PASP, mmHg	79.19 ± 26.96	83.01 ± 24.89	78.34 ± 27.36	0.190
TAPSE, mm	16.74 ± 3.92	16.05 ± 3.90	16.89 ± 3.93	0.139
TAPSE/PASP, mm/mmHg	0.23 ± 0.12	0.21 ± 0.09	0.24 ± 0.13	**0.036**
Pericardial effusion, n (%)	81 (20.4)	24 (33.3)	57 (17.5)	**0.003**
*Right heart catheterization*				
S_v_O_2_, %	66.03 ± 7.61	63.00 ± 9.48	66.70 ± 6.97	** < 0.001**
RAP, mmHg	6.52 ± 3.75	7.72 ± 4.54	6.25 ± 3.51	**0.003**
mPAP, mmHg	48.60 ± 3.76	50.19 ± 17.92	48.24 ± 18.66	0.419
PAWP, mmHg	8.89 ± 3.37	9.32 ± 3.44	8.79 ± 3.35	0.229
CI, L·min·m^−2^	2.70 ± 0.97	2.43 ± 0.82	2.77 ± 0.99	**0.008**
PVR, Wood units	10.76 ± 6.84	11.89 ± 7.78	10.50 ± 6.61	0.120
*Laboratory test*				
NT-proBNP, pg/mL	619.50 (196.75–1629.75)	1212.50(564.75–2393.50)	507.00(177.25–1447.50)	** < 0.001**
BUN, mmol/L	6.82 ± 2.36	7.01 ± 2.46	6.78 ± 2.34	0.470
Scr, μmoI/L	81.24 ± 18.01	85.96 ± 18.25	79.71 ± 17.80	**0.006**
Cystatin C, mg/L	0.97 ± 0.29	1.08 ± 0.33	0.95 ± 0.28	**0.001**
*Treatment*				
PAH-targeted therapy ^b^, n (%)	315 (79.1)	58 (80.6)	257 (78.8)	0.745
Combination therapy, n (%)	181 (45.5)	21 (29.2)	160 (49.1)	**0.002**

*BMI* Body mass index, *BUN* Blood urea nitrogen, *CHD* Congenital heart disease, *CI* Cardiac index, *CTD* Connective tissue disease, *CTEPH* Chronic thromboembolism pulmonary hypertension, *CW* Clinical worsening, *IPAH* Idiopathic pulmonary hypertension, *LVDd* Left ventricular end-diastolic diameter, *LVEF* Left ventricular ejection fraction, *mPAP* Mean pulmonary arterial pressure, *NT-proBNP* N-terminal pro-brain natriuretic peptide, *PAH* Pulmonary arterial hypertension, *PASP* Pulmonary arterial systolic pressure, *PAWP* Pulmonary arterial wedge pressure, *PVR* Pulmonary vascular resistance, *RAP* Right atrium pressure, *RVDd* Right ventricular end-diastolic diameter, *Scr* Serum creatinine, *S*_*v*_*O*_*2*_ Mixed venous oxygen saturation, *TAPSE* Tricuspid annular plane systolic excursion, *WHO-FC* World Health Organization functional class, *6MWD* 6-min walking distance

^a^Others pre-capillary PH subtype include heritable PAH (*n* = 15), drug and toxin-induced PAH (*n* = 3), portal hypertension-associated PAH (*n* = 4), PVOD/PCH (*n* = 6), and other pulmonary artery obstructions (arteritis and congenital pulmonary arteries stenoses) (*n* = 28), PH with unclear and/or multifactorial mechanisms (*n* = 16)

^b^PAH-targeted therapy included endothelin receptor antagonists, nitric oxide-cGMP enhancers and prostacyclin pathway agonists

During a median follow-up of 282 (187–377) days, 72 (18.1%) patients experienced clinical worsening, including 11 deaths and 61 rehospitalizations for HF or deterioration of PH. Patients who experienced clinical worsening had significantly more impaired renal function compared with patients without clinical worsening, as evidenced by elevated cystatin C (1.08 ± 0.33 mg/L vs. 0.95 ± 0.28 mg/L, *P* = 0.001), serum creatinine (85.96 ± 18.25 μmoI/L vs. 79.71 ± 17.80 μmoI/L, *P* = 0.006), and eGFR derived from different equations (all *P* < 0.05) (Table 2).

**Table 2:** Baseline eGFR and eGFR stages classification

Variables	Total (*N* = 398)	CW (*n* = 72)	No-CW (*n* = 326)	*P*-value
CKD stages, n (%)^a^				**0.010**
Stage 1	159 (39.9)	17 (23.6)	142 (43.6)	
Stage 2	186 (46.7)	41 (56.9)	145 (44.5)	
Stage 3	51 (12.8)	14 (19.4)	37 (11.3)	
Stage 4	2 (0.5)	0 (0.0)	2 (0.6)	
eGFR, mL/min/1.73m^2^				
2009 CKD-EPI_cr_	80.64 ± 21.93	76.00 ± 22.14	81.67 ± 21.79	**0.047**
2012 CKD-EPI_cys_	81.94 ± 26.34	73.35 ± 26.74	83.75 ± 25.89	**0.002**
2012 CKD-EPI_cr-cys_	81.10 ± 22.82	74.21 ± 23.24	82.62 ± 22.47	**0.005**
2021 CKD-EPI_cr_	84.61 ± 22.06	80.19 ± 22.45	85.59 ± 21.89	0.060
2021 CKD-EPI_cr-cys_	84.15 ± 23.10	77.12 ± 23.69	85.71 ± 22.71	**0.004**
MDRD	78.57 ± 22.16	74.72 ± 22.06	79.42 ± 22.13	0.103
Cockcroft-Gault	78.96 ± 36.34	75.31 ± 38.18	79.77 ± 35.95	0.347

*CKD* Chronic kidney disease, *CKD-EPI* CKD-Epidemiology Collaboration, *Cr* Creatinine, *CW* Clinical worsening, *Cys* Cystatin C, *eGFR* Estimated glomerular filtration rate measured in ml/min per 1.73 m^2^, *MDRD* Modification of Diet in Renal Disease

^a^eGFR stages were determined according to Kidney Disease Improving Global Outcomes guidelines. Baseline GFR was estimated using different equations

### Univariable and multivariable Cox regression

**Fig. 1:**
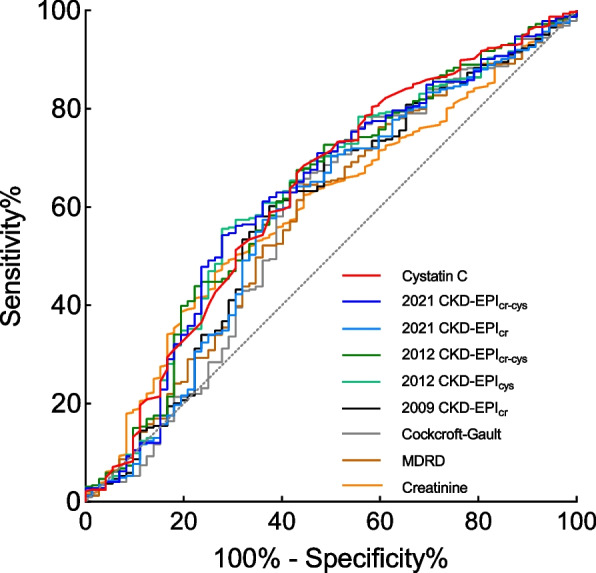
Receiver operator curves for parameters and eGFR derived from different equations. CKD-EPI, Chronic Kidney Disease-Epidemiology Collaboration; Cr, creatinine; Cys, cystatin C; eGFR, estimated glomerular filtration rate measured in ml/min per 1.73 m^2^; MDRD, Modification of Diet in Renal Disease. Depicts the receiver operator curves illustrating the performance of cystatin C, creatinine and estimated glomerular filtration rate derived from different equations

On univariable analysis, predictors of outcome were shown in Table S2, including age, WHO-FC, combined targeted therapy, RAP, cardiac index, SvO_2_, NT-proBNP, creatinine and cystatin C (all *P* < 0.05). ROC curves analysis illustrated the discriminative capacity of different parameters of renal function (Fig. 1). Cystatin C had the highest AUC for outcome prediction (0.630; 95% confidence interval [CI]: 0.556 to 0.704; *P* < 0.001), followed by 2021 CKD-EPI eGFR_cr-cys_ (AUC: 0.625; 95% CI: 0.551 to 0.699) (Table S1).

On multivariable Cox analyses, Cystatin C remained independently associated with clinical worsening after adjusting for age, female, PH subtype and combined targeted therapy [hazard ratio (HR) 2.184, 95% CI 1.200–3.975, *P* = 0.011] in Model 1, after adjusting for invasive hemodynamic parameters including RAP, SvO_2_ and cardiac index [HR 2.212, 95% CI 1.184–4.131, *P* = 0.013] in Model 2, and after adjusting WHO-FC and NT-proBNP [HR 2.266, 95% CI 1.210–4.244, *P* = 0.011] in Model 3 (Table 3).

**Table 3:** Multivariate Cox regression models of cystatin C and prognosis

Variables	HR	95% CI	*P*-value
*Model 1*			
Cystatin C	2.184	1.200–3.975	**0.011**
Age	0.998	0.980–1.015	0.998
Female	1.294	0.799–2.095	0.295
PH subtype	0.797	0.567–1.121	0.192
Combined target therapy	0.462	0.245–0.872	**0.017**
*Model 2*			
Cystatin C	2.212	1.184–4.131	**0.013**
RAP	1.070	1.015–1.129	**0.012**
S_v_O_2_	0.962	0.928–0.998	**0.037**
CI	0.847	0.606–1.184	0.332
*Model 3*			
Cystatin C	2.266	1.210–4.244	**0.011**
WHO-FC: III or IV/I or II	2.041	1.230–3.388	**0.006**
NT-proBNP	1.000	1.000–1.000	0.420
*Model 4*			
Cystatin C	2.080	1.085–3.985	**0.027**
Swedish/COMPERA	2.483	1.646–3.744	** < 0.001**

Four models were constructed to adjust clinical features, hemodynamic parameters, non-invasive parameters and Swedish/COMPERA risk stratification, respectively

*CI* Cardiac index, *COMPERA* Comparative, Prospective Registry of Newly Initiated Therapies for Pulmonary Hypertension, *NT-proBNP* N-terminal pro-brain natriuretic peptide, *PH* Pulmonary hypertension, *RAP* Right atrium pressure, *S*_*v*_*O*_*2*_ Mixed venous oxygen saturation, *WHO-FC* World Health Organization functional class

As shown in Figure S2, a restricted cubic spline confirmed the linear relationship between cystatin C and clinical worsening (Nonlinear *P* = 0.164). ROC curve analysis revealed that when the cut-off value was set at 1.0 mg/L, cystatin C displayed the best predictive value with a sensitivity of 55.6% and a specificity of 68.4%. Accordingly, patients were further stratified into two groups based on the optimal cut-off of cystatin C. Kaplan–Meier event-free survival curves confirmed that patients with cystatin C ≥ 1.0 mg/L had a higher risk than those with a cystatin C < 1.0 mg/L (Fig. 3, A).

### Relationship between cystatin C, clinical and hemodynamic parameters

**Fig. 2:**
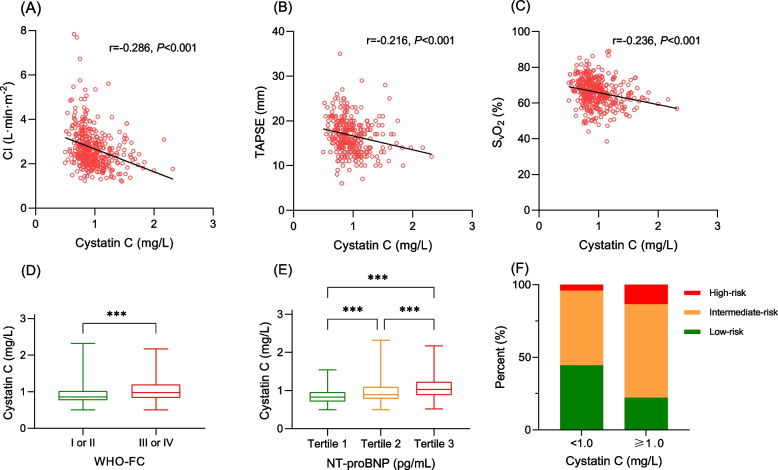
Relationship between cystatin C and disease severity. **A** Correlation (Spearman) with cardiac index, **B** Correlation (Spearman) with TAPSE, **C** Correlation (Spearman) with SvO_2_, **D** Cystatin C in different WHO-FC (***P < 0.001), **E** NT-proBNP (stratified by tertile) and cystatin C (***P < 0.001), **F** Swedish/COMPERA risk stratification and cystatin C. CI, cardiac index; COMPERA, Comparative, Prospective Registry of Newly Initiated Therapies for Pulmonary Hypertension; NT-proBNP, N-terminal pro-brain natriuretic peptide; SvO_2_, mixed venous oxygen saturation; TAPSE, tricuspid annular plane systolic excursion; WHO-FC, World Health Organization functional class. Presents the relationship between cystatin C levels and disease severity in the context of hemodynamic status, functional class, NT-proBNP and risk stratification

Regarding clinical variables, there was a direct correlation between cystatin C levels and the World Health Organization functional class (*P* < 0.001), as well as NT-proBNP levels (*P* < 0.001). While there were some weak linear correlations observed between cystatin C and Cardiac index (*r* = -0.286, *P* < 0.001), SvO_2_ (*r* = -0.216, *P* < 0.001), and TAPSE (*r* = -0.236, *P* < 0.001), it was found that cystatin C levels increased as cardiac index, SvO_2_, and TAPSE decreased (as illustrated in Fig. 2, A-E).

### Comparison of invasive and non-invasive risk assessment strategies

**Fig. 3:**
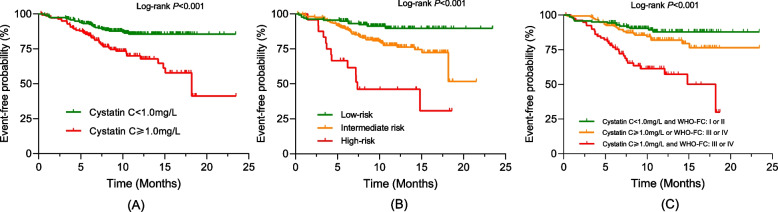
Kaplan–Meier analysis for the probability of endpoint events. **A** Patients stratified by the cystatin C cut-off; **B** Patients stratified by the Swedish/COMPERA risk model; **C** Patients stratified by the combination of cystatin C and World Health Organization functional class. Presents the Kaplan–Meier analysis, which illustrates the probability of endpoint events stratified by cystatin C cut-off (1.0 mg/L), Swedish/COMPERA risk model and the combination of cystatin C and WHO-FC

**Fig. 4:**
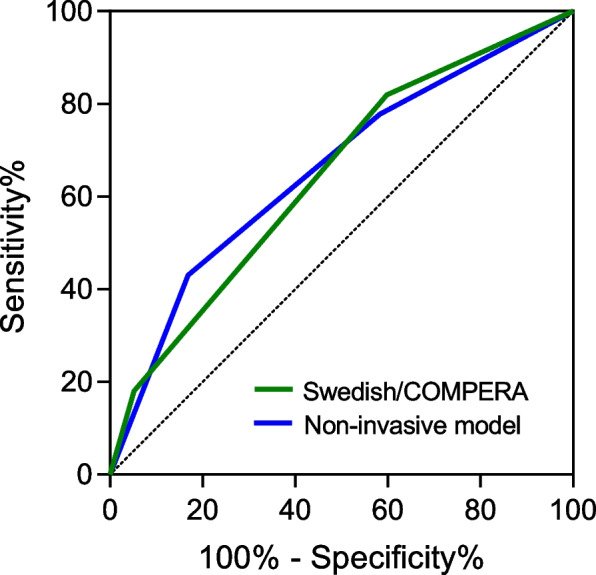
Area under the curve for clinical worsening using the Swedish/COMPERA and the non-invasive risk stratification strategy. COMPERA, Comparative, Prospective Registry of Newly Initiated Therapies for Pulmonary Hypertension. DeLong test pairwise comparison: Non-invasive model vs. Swedish/COMPERA: Area under the curve 0.657 vs 0.643, *P* = 0.619. Showcases the area under the curve analysis for clinical worsening, comparing the predictive capacity of Swedish/COMPERA risk stratification strategy with a non-invasive risk stratification model

After stratifying patients based on invasive risk stratification (using Swedish/COMPERA), 144 (36.2%) patients were classified as low-risk, 224 (56.3%) as intermediate-risk, and 30 (7.5%) as high-risk categories. Patients with higher cystatin C values had a greater proportion classified as high-risk than patients with lower cystatin C values (Fig. 2, F), and patients in high-risk group had significantly poorer outcomes compared with other risk groups (Fig. 3, B). Additionally, patients were divided into three groups using the dichotomous cut-off value for both WHO-FC and cystatin C: low-risk: both WHO-FC (I or II) and cystatin C (< 1.0 mg/L); high-risk: advanced WHO-FC (III or IV) and elevated cystatin C (≥ 1.0 mg/L); and intermediate-risk (the remaining patients). Kaplan–Meier analysis revealed good discrimination power among patients in different risk groups, as demonstrated in Fig. 3, C. Furthermore, we compared the ROC curves for prognosis predicting between Swedish/COMPERA stratification and our non-invasive model. The predictive power of our noninvasive models was comparable to that of the invasive model (AUC 0.657 vs 0.643, *P* = 0.619) (Fig. 4).

## Discussion

The main findings of the current study can be succinctly stated as follows: (1) Among the various markers and equations regarding renal function, cystatin C and its derived equations demonstrate favorable predictive value for clinical worsening; (2) Cystatin C was correlated with traditional prognostic parameters, such as WHO-FC, NT-proBNP levels, cardiac index, SvO_2_ and TAPSE; (3) Baseline cystatin C closely linked to the risk of clinical worsening, independent of demographic parameters and RHC-derived variables; Cystatin C ≥ 1.0 mg/L discriminated a group of patients with a higher risk of clinical worsening; 4) A non-invasive risk model incorporating cystatin C and WHO-FC exhibited a predictive value comparable to that of invasive risk assessment recommended by guidelines. Our findings suggested that cystatin C served an important role in the non-invasive identification of high-risk patients with pre-capillary PH.

Renal dysfunction is a common comorbidity in patients with PH. The potential mechanisms through which PH impacts the renal function include (1) altered cardiac and/or renal hemodynamics (including increased venous congestion, reduced cardiac output, et al.) [21]; (2) neurohormonal activation [22]; and (3) oxidative stress and inflammatory response. In cases of ineffective pumping by the right ventricle, elevated central venous pressure can impede venous return, leading to venous congestion in kidneys, resulting in reduced renal blood flow and ultimately, the development of renal dysfunction. Additionally, PH is characterized by decreased cardiac output, which subsequently diminishes organ perfusion, including that of the kidneys, resulting in a decreased eGFR [23, 24]. Moreover, there is emerging evidence suggesting that a combination of PH and chronic kidney disease can trigger increased neurohormonal activation, which might be linked to exacerbating vascular remodeling in both the pulmonary and renal circulations. Lastly, chronic inflammation is a hallmark of HF as commonly seen in PH and may contribute to the development and progression of renal dysfunction. Inflammation leads to the release of pro-inflammatory cytokines, chemokines, and reactive oxygen species, which contribute to the oxidative stress. In turn, subsequent endothelial dysfunction, glomerular damage, and tubular injury occurs, ultimately impairing renal function [25, 26].

Data from an updated REVEAL cohort reported a prevalence of renal dysfunction defined by GFR < 60 mL/min/1.73 m^2^ in 29% of PAH patients [21]. With the increasing age at diagnosis of pre-capillary PH population, the comorbidity burden has continued to rise. Recent data from the Italian PATRIARCA Registry, a multi-center cohort investigating elderly pre-capillary PH patients, showed that kidney dysfunction was prevalent in 37% of patients aged 70 years or older [27]. Notably, previous studies mainly calculated eGFR via creatinine-based equations. However, creatinine is affected by factors such as age, muscle mass and diet, and is thus a less sensitive marker of renal function compared to cystatin C [28]. Studies have shown that equations combining both creatinine and cystatin C were more accurate in estimating GFR, especially the new race-free 2021 CKD-EPI equation [11]. In this study, we adopted the 2021 CKD-EPI_cr-cys_ equation, and consistent with previous studies, we observed that PH patients were at a relatively high risk of coexisting kidney dysfunction. The prevalence of kidney dysfunction was 13.3%, 11.4% and 9.6% in patients with pre-capillary PH, PAH and CTEPH (defined by GFR < 60 mL/min/1.73 m^2^), respectively, and increased to 40.4% in individuals aged over 65 years. The decline in eGFR profoundly impacted outcomes in PH, with disease severity and mortality increasing in patients with advanced CKD stage [21, 29, 30].

Although cystatin C is increasingly utilized for GFR estimation, cystatin C per se may be a better indicator in clinical practice. The first reason lies in its simplicity and convenience. Secondly, cystatin C is a more comparable index across studies than eGFR, as the latter is calculated by different equations. Thirdly, as shown in our study, the predictive power of cystatin C is comparable to that of eGFR; Lastly, cystatin C not only reflects renal function, but also links to chronic inflammation and oxidization stress. The elevation of cystatin C has been observed in patients with cardiovascular diseases including pre-capillary PH. In our study, patients with higher cystatin C levels tended to be older, predominantly male, and have advanced WHO-FC, more comorbidities, higher NT-proBNP levels and worse heart function. Significant correlations were observed between cystatin C and cardiac index and TAPSE, which are well-established markers of disease severity. These findings suggested that cystatin C is a promising biomarker for patients with pre-capillary PH. However, the association between cystatin C and RAP did not show significance (*r* = 0.083, *P* = 0.100) (Figure S3), though RAP is closely connected with venous congestion in kidneys. These findings are consistent with several previous studies that found no correlations between eGFR with RAP in patients with PH [6]. Nevertheless, to gain a comprehensive understanding of the relationship between hemodynamic status in PH and renal function, further investigations are warranted.

Consistent with our results, several studies have highlighted the utility of cystatin C for non-invasive PH risk assessment. The impairment of renal function markers including cystatin C was proved to be associated with significantly lower long-term survival rates in 64 patients with pre-capillary PH [6]. Similarly, in a prospective study of 59 congenital heart disease-associated PAH patients, cystatin C predicted long-term mortality and clinical worsening [4]. In a small cohort of 14 PAH patients, cystatin C was abnormally elevated compared with controls and correlated with RV morphology, function and pressure abnormalities obtained by echocardiography and cardiac magnetic resonance imaging [5]. Furthermore, a recent study conducted on PH children observed a significant positive correlation between cystatin C and the right ventricle Tei index [7]. Concerning diseases associated with PH, Selvaraj et al. [30] demonstrated that Log cystatin C was directly correlated with echocardiogram-derived PASP in patients with chronic kidney disease; Secemsky et al. [31] analyzed 332 HIV-infected patients and found that elevated cystatin C was associated with the development of PH; for patients with systemic lupus erythematosus, cystatin C differentiated those with severe cardiovascular prognosis [32]; and in HF patients, cystatin C independently predicted outcome [33].

These findings suggested that cystatin C is a valuable biomarker for PH across of various groups and etiologies. However, there is a lack of data regarding the cut-off value of cystatin C for predicting outcomes in PH. One study has demonstrated PAH patients with cystatin C > 1.10 mg/L showed a significantly higher mortality rate [4]. Another study stratified pre-capillary PH patients by cystatin C at an optimized cut-off of 1.0 mg/L [6]. Close to previously established thresholds, in our study, the ROC curve demonstrated an optimal cystatin C cut-off of ≥ 1.0 mg/L for predicting clinical worsening. Thus, it is recommended that clinical physicians closely monitor patients with high cystatin C levels to provide vigilant monitoring, timely interventions, and personalized care, thereby preventing potential deteriorations in their condition.

Despite increasing evidence supporting the role of cystatin C in prognosis prediction, current risk stratification algorithms recommended by guidelines or applied in clinical practice do not take cystatin C into account. Kidney dysfunction is incorporated in the REVEAL Risk Score Calculator 2.0, but the biomarker used in GFR estimation is undefined, and the Calculator is relatively complex. In our study, we categorized patients using Swedish/COMPERA models, and found that cystatin C remained an independent predictor of clinical worsening after adjusting for current risk stratification. Additionally, we built a non-invasive risk model incorporating cystatin C and WHO-FC, and our results showed that the c-statistic of noninvasive model was comparable to that of Swedish/COMPERA. While our study highlights the potential significance of cystatin C as a non-invasive prognostic marker, it is essential to acknowledge the limitations inherent in the observed correlations and the moderate predictive capacity of the developed risk model. Future research and validation studies involving larger cohorts and comparisons with established risk models are warranted to further ascertain the clinical utility of cystatin C in prognostic evaluation. The present findings, although promising, should be interpreted with caution and considered as an initial step towards uncovering the potential value of cystatin C in enhancing non-invasive prognostic assessments in the field of PH.

Our study represents the largest study to date of the relationship between cystatin C and pre-capillary PH. We compared the prognostic value of cystatin C with other biomarkers and eGFR equations, and we highlighted the potential for cystatin C to enhance non-invasive risk stratification. However, several potential limitations should also be addressed. Firstly, due to the observational design of our study, further studies are warranted to unveil the underlying mechanisms between cystatin C and PH. Secondly, according to the Kidney Dialysis Outcomes Quality Initiative CKD definition guidelines, two separate measurements of eGFR taken at least three months apart are required for robust evaluation of renal function impairment. Unfortunately, our data only included a single measurement of cystatin C and creatinine. Thirdly, in patients with HF, aberrations in body composition may affect cystatin C levels and potentially decrease its accuracy in GFR estimation, therefore, our results should be interpreted cautiously [34]. Lastly, the study population of pre-capillary PH comprises a heterogeneous etiology, and patients with different underlying causes may require varying treatment strategies. For instance, some CTEPH patients undergo interventions or surgical treatments, which could potentially impact the interpretation of the outcomes.

## Conclusion

In this study, we comprehensively investigated various markers and equations of renal function in the context of pre-capillary PH, and we have identified cystatin C as a crucial parameter for assessing disease severity and predicting prognosis. Importantly, our findings shed light on the previously unexplored application of cystatin C in conjunction with WHO functional class for non-invasive risk stratification, demonstrating comparable predictive efficacy with established invasive risk stratification models. The non-invasive nature of cystatin C assessment further enhances its clinical value, offering a valuable tool for guiding goal-oriented treatment strategies.

### Supplementary Information

**Additional file 1: Figure S1.** Flowchart. Legends: HF, heart failure; PH, pulmonary hypertension; RHC, right heart catheterization. **Figure S2.** Restricted cubic spline of cystatin C levels for the risk of clinical worsening. Legend: HR, hazard ratio. **Figure S3.** Relationship between cystatin C and RAP. Legend: RAP, right atrial pressure. **Table S1.** The Scoring of the Swedish/COMPERA Prediction Model. **Table S2.** Univariable Cox analysis for clinical worsening prediction. **Table S3. **ROC Curve Analysis for renal function parameters in Predicting Clinical Worsening.

## Data Availability

The datasets used during the current study are available from the corresponding author on reasonable request.

## Abbreviations

AUC: Area under the curve
CKD-EPI: Chronic Kidney Disease Epidemiology Collaboration
GFR: Glomerular filtration rate
HF: Heart failure
MDRD: Modification of Diet in Renal Disease
mPAP: Mean pulmonary artery pressure
NT-proBNP: N-terminal pro-brain natriuretic peptide
PAH: Pulmonary arterial hypertension
PASP: Systolic pulmonary artery pressure
PH: Pulmonary hypertension
RAP: Right atrial pressure
RHC: Right heart catheterization
ROC: Receiver operating characteristic
SvO_2_: Mixed venous oxygen saturation
TAPSE: Tricuspid annular plane systolic excursion
WHO-FC: World Health Organization functional class
6MWD: 6-Minute walking distance
