# Supplementary material for: The potential of cystatin C as a predictive biomarker in pulmonary hypertension
Source: BMC Pulm Med. 2023 Aug 26;23:311. doi: 10.1186/s12890-023-02595-1 (PMC10463899; doi:10.1186/s12890-023-02595-1)
Supplement: Supplementary file 1 — Additional file 1: Figure S1. Flowchart. Legends: HF, heart failure; PH, pulmonary hypertension; RHC, right heart catheterization. Figure S2. Restricted cubic spline of cystatin C levels for the risk of clinical worsening. Legend: HR, hazard ratio. Figure S3. Relationship between cystatin C and RAP. Legend: RAP, right atrial pressure. Table S1. The Scoring of the Swedish/COMPERA Prediction Model. Table S2. Univariable Cox analysis for clinical worsening prediction. Table S3. ROC Curve Analysis for renal function parameters in Predicting Clinical Worsening. [file 12890_2023_2595_MOESM1_ESM.doc]

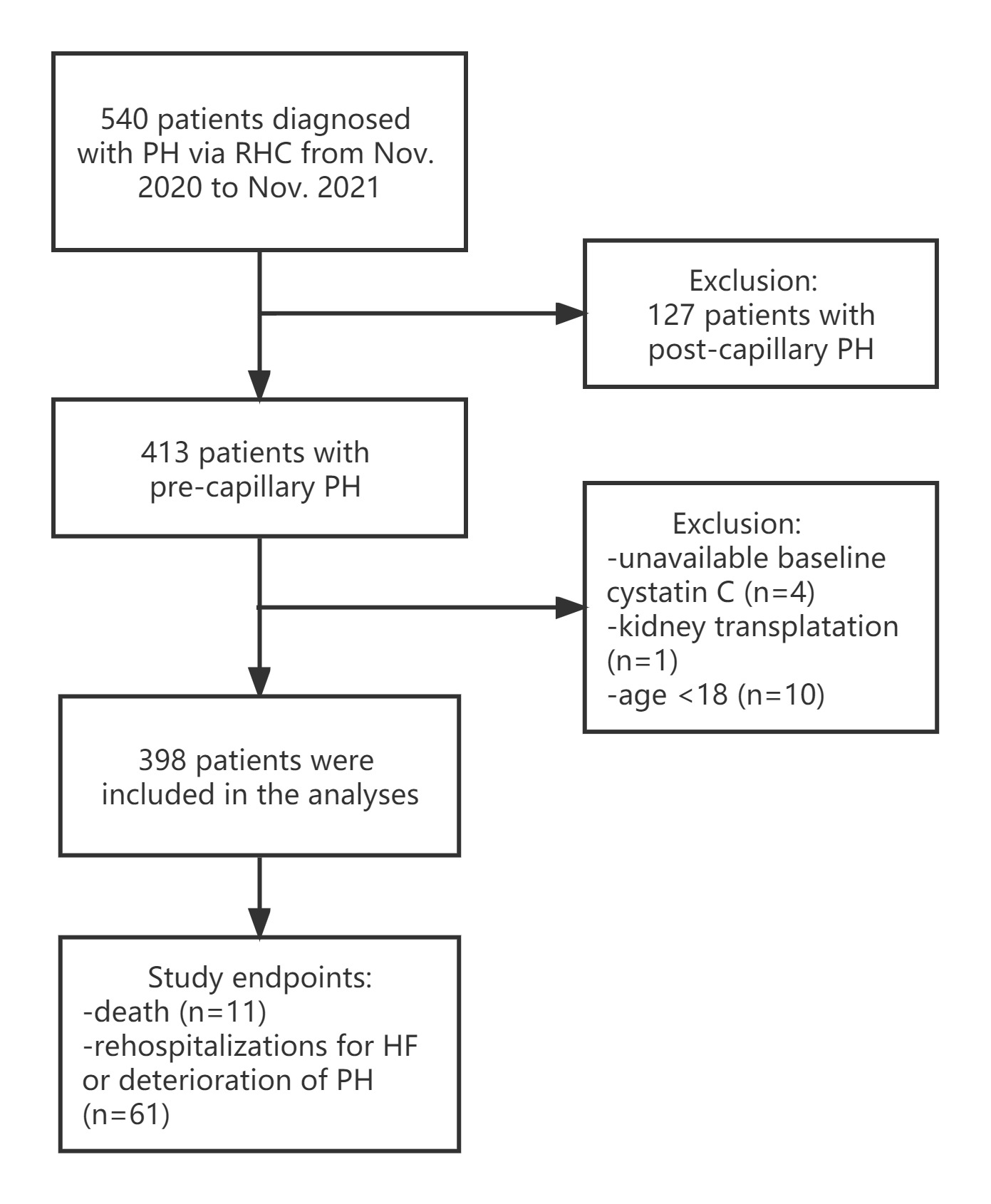


Figure S1

Title: Flowchart

Legends: HF, heart failure; PH, pulmonary hypertension; RHC, right heart catheterization.


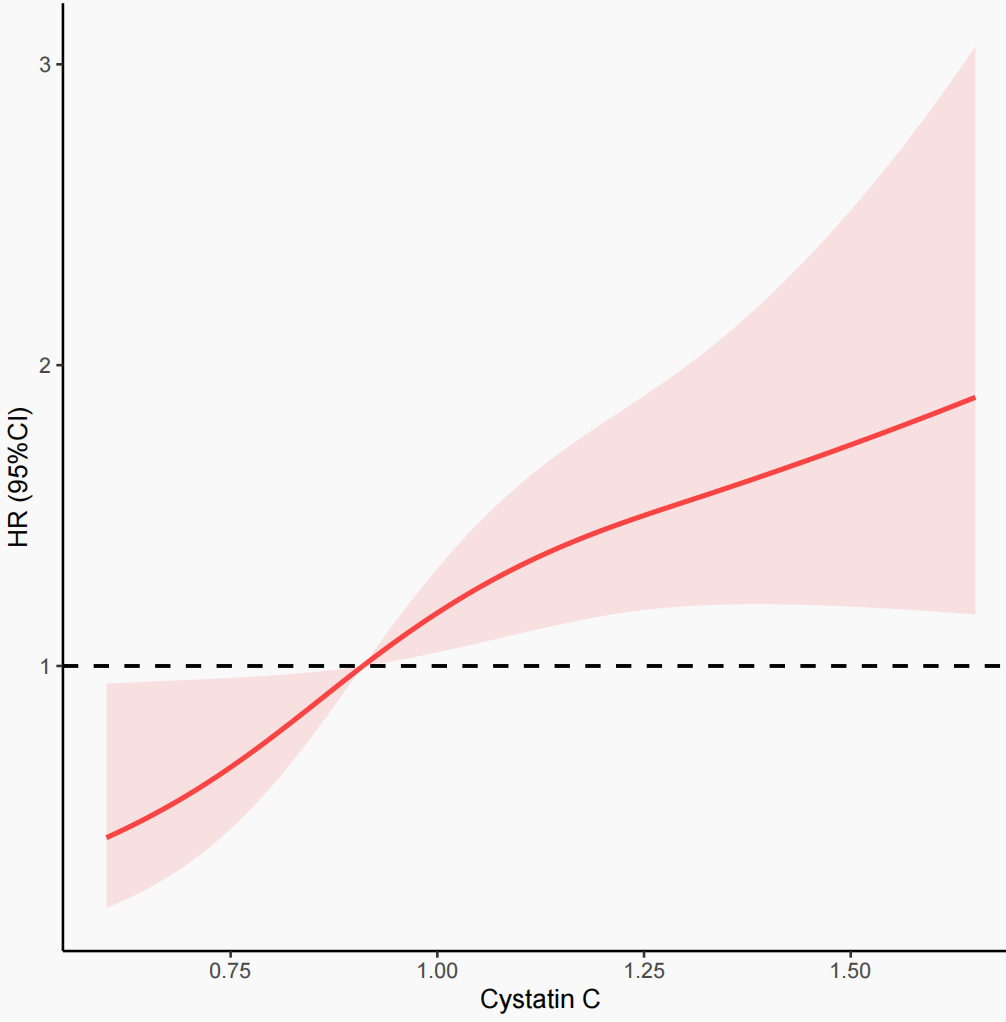


Figure S2

Title: Restricted cubic spline of cystatin C levels for the risk of clinical worsening.

Legend: HR, hazard ratio.


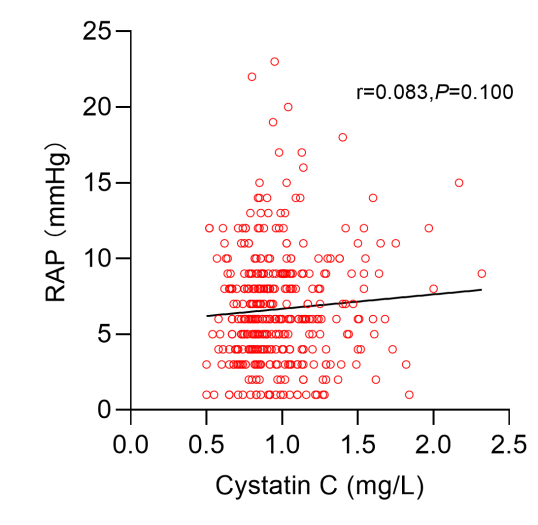


Figure S3

Title: Relationship between cystatin C and RAP.

Legend: RAP, right atrial pressure.

**Table S1** The Scoring of the Swedish/COMPERA Prediction Model

| Points assigned | 1 | 2 | 3 |
| --- | --- | --- | --- |
| WHO-FC | I/II | III | IV |
| 6MWD | >440 m | 440-165 m | <165 m |
| NT-proBNP  or  BNP | <300 pg/mL  <50 pg/mL | 300-1100 pg/mL  <50-800 pg/mL | >1100 pg/mL  >800 pg/mL |
| RAP | <8 mmHg | 8-14 mmHg | >14 mmHg |
| CI | ≥2.5 L·min^-1^·m^-2^ | 2.0-2.5 L·min^-1^·m^-2^ | <2.0 L·min^-1^·m^-2^ |
| SvO_2_ | >65% | 60-65% | <65% |

BNP, brain natriuretic peptide; CI, cardiac index; NT-proBNP, N-terminal pro-brain natriuretic peptide; RAP, right atrial pressure; SvO_2_, mixed venous oxygen saturation; WHO-FC, World Health Organization function class; 6MWD, 6-minute walk distance.

**Table S2.** Univariate Cox analysis for clinical worsening prediction.

| Variables | Wald | HR | 95% CI | *P*-value |
| --- | --- | --- | --- | --- |
| Age | 4.17 | 1.014 | 1.001-1.028 | **0.041** |
| Female sex | 3.47 | 0.643 | 0.404-1.023 | 0.062 |
| Body mass index | 1.38 | 0.968 | 0.918-1.022 | 0.240 |
| WHO-FC | 11.81 | 2.055 | 1.363-3.100 | **0.001** |
| 6 minute walk distance | 0.01 | 1.000 | 0.996-1.004 | 0.934 |
| Hypertension | 0.00 | 1.008 | 0.584-1.738 | 0.978 |
| Diabetes mellitus | 1.46 | 1.509 | 0.773-2.943 | 0.228 |
| Dyslipidemia | 0.03 | 0.939 | 0.450-1.958 | 0.866 |
| *Hemodynamic parameters* | | | | |
| Right atrium pressure | 9.32 | 1.087 | 1.030-1.146 | **0.002** |
| mPAP | 0.51 | 1.004 | 0.992-1.017 | 0.476 |
| PAWP | 1.30 | 1.041 | 0.972-1.114 | 0.255 |
| PVR | 2.12 | 1.024 | 0.992-1.058 | 0.145 |
| SvO_2_ | 15.56 | 0.941 | 0.913-0.970 | **<0.001** |
| Cardiac index | 6.69 | 0.644 | 0.461-0.899 | **0.010** |
| *Laboratory test* | | | | |
| NT-proBNP^a^ | 6.81 | 0.232 | 1.261-1.059 | **0.009** |
| Blood urea nitrogen | 0.87 | 1.046 | 0.951-1.151 | 0.351 |
| Serum creatinine | 8.16 | 1.016 | 1.005-1.027 | **0.004** |
| Cystatin C | 12.30 | 2.638 | 1.534-4.537 | **<0.001** |
| *Treatment* | | | | |
| PAH-specific therapy | 0.33 | 1.186 | 0.661-2.129 | 0.568 |
| Combination therapy | 7.53 | 0.491 | 0.295-0.816 | **0.006** |

^a^Per standard deviation increase.

mPAP, mean pulmonary arterial pressure; NT-proBNP, N-terminal pro-brain natriuretic peptide; PAH, pulmonary arterial hypertension; PAWP, pulmonary arterial wedge pressure; PVR, pulmonary vascular resistance; SvO_2_, mixed venous oxygen saturation; WHO-FC, World Health Organization functional class.

**Table S3.** ROC Curve Analysis for renal function parameters in Predicting Clinical Worsening

| Variable | Clinical worsening | | |
| --- | --- | --- | --- |
|  | AUC (95%CI) | Sensitivity/Specificity | *P-*value |
| Cystatin C | 0.630 (0.556-0.704) | 55.56/68.40 | **<0.001** |
| Creatinine | 0. 619 (0.529-0.666) | 72.22/52.78 | **0.008** |
| eGFR |  |  |  |
| 2021 CKD-EPI_cr_ | 0.586 (0.509-0.663) | 58.33/63.89 | **0.029** |
| 2021 CKD-EPI_cr-cys_ | 0.625 (0.551-0.699) | 72.22/54.32 | **0.001** |
| 2012 CKD-EPI_cr-cys_ | 0.625 (0.550-0.699) | 56.94/67.48 | **0.001** |
| 2012 CKD-EPI_cys_ | 0.623 (0.549-0.697) | 72.22/55.56 | **0.001** |
| 2009 CKD-EPI_cr_ | 0.591 (0.514-0.667) | 65.28/58.02 | **0.021** |
| MDRD | 0.581 (0.505-0.656) | 58.33/63.89 | **0.029** |
| Cockcroft-Gault | 0.578 (0.499-0.657) | 59.72/62.96 | 0.054 |

CKD-EPI, CKD-Epidemiology Collaboration; Cr, creatinine; Cys, cystatin C; eGFR, estimated glomerular filtration rate measured in ml/min per 1.73 m^2^; MDRD, Modification of Diet in Renal Disease.
